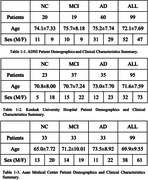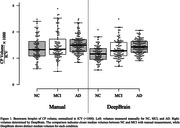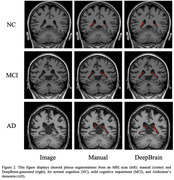# Quantitative Analysis of Choroid Plexus Enlargement in Alzheimer's Dementia: A Study of Automated Volumetric Technique

**DOI:** 10.1002/alz.088824

**Published:** 2025-01-09

**Authors:** Dong‐Hee Kim, Chong Hyun Suh, Wooseok Jung, Seung Hyun Lee, Jinyoung Kim, Hyeonwoo Cho, Yeha Lee, Sang Joon Kim, Won‐Jin Moon

**Affiliations:** ^1^ VUNO Inc., Seoul Korea, Republic of (South); ^2^ Asan Medical Center, University of Ulsan College of Medicine, Seoul Korea, Republic of (South); ^3^ Konkuk University School of Medicine, Seoul Korea, Republic of (South)

## Abstract

**Background:**

Choroid plexus enlargement is implicated in the exacerbation of cognitive impairments characteristic of Alzheimer’s dementia (AD). Manual volumetric assessment by radiologists, while accurate, is impractical for large‐scale application due to its resource‐intensive nature. We examine the use of automated brain volumetry software as an efficient and cost‐effective alternative for quantifying choroid plexus volume.

**Method:**

We collected 3D T1 brain MRI images from 293 patients aged between 44 and 91, with a median age of 73, at Konkuk University Hospital (n = 95), Asan Medical Center (n = 99), and the Alzheimer’s Disease Neuroimaging Initiative (ADNI) (n = 99). The study comprised 76 normal control (NC), 89 mild cognitive impairment (MCI), and 128 AD cases, with a female majority of 58.4%. Using VUNO‐Med DeepBrain software, we obtained choroid plexus segmentation masks and volumes from both radiologists and neural networks trained with deep learning. All choroid plexus volumes were normalized by intracranial volume (ICV) multiplied by 1000. Additionally, to assess DeepBrain's accuracy, we calculated the Intraclass Correlation Coefficient (ICC(2, k)) between the manually labeled volumes and those labeled by DeepBrain.

**Result:**

The median choroid plexus volumes measured by radiologists were 1.328 for NC, 1.354 for MCI, and 1.495 for AD. In contrast, measurements by DeepBrain were 1.163 (NC), 1.284 (MCI), and 1.428 (AD). A one‐way ANOVA analysis (NC vs. MCI vs. AD) revealed that DeepBrain‐based measurements (p < 0.001) may be more significant than manual measurements (p = 0.17) in elucidating the relationship between choroid plexus enlargement and cognitive impairments in AD. Additionally, the Intraclass Correlation Coefficient (ICC(2, k)) between manual and DeepBrain volumes indicated a moderate correlation with a score of 0.69 (p < 0.001).

**Conclusion:**

Our results suggest that automated brain volumetry is a reliable method for assessing choroid plexus volume, correlating with the cognitive impairment continuum in AD. Its implementation could significantly streamline the diagnostic process, offering a scalable and objective approach for clinicians and researchers.